# A Case of a COVID-19-positive Patient

**DOI:** 10.7759/cureus.7608

**Published:** 2020-04-09

**Authors:** Zohra R Malik, Zareen Razaq, Nassim Mokraoui, Tomasz Zrodlowski, Snehal Bansod

**Affiliations:** 1 Internal Medicine, St. John's Episcopal Hospital, Far Rockaway, USA; 2 Internal Medicine, Ghurki Trust Teaching Hospital, Lahore, PAK

**Keywords:** covid-19, cough, fever, chills, fibrosis, ground-glass opacity, chloroquine, coronavirus

## Abstract

The coronavirus (COVID-19), discovered in 2019, has been creating havoc since it first emerged in China and is now spreading worldwide. Its presentation is somewhat similar to influenza. We hereby discuss the salient features of the coronavirus and present the case of a 33-year-old male who was tested positive for COVID-19.

## Introduction

Virus SARS-CoV-2 (severe acute respiratory syndrome coronavirus-2) or the 2019 novel coronavirus (2019-nCoV) belong to the broad family of coronaviruses (subgenus Sarbecovirus). Viruses use a nested set of messenger ribonucleic acids (mRNAs) to replicate. There are four genera of coronaviruses: alpha, beta, gamma, and delta. Two of these genera are known to infect humans: alpha coronaviruses (the human coronavirus 229E (HCoV-229E) and human coronavirus NL63 (HCoV-NL63)) and beta coronaviruses (human coronavirus HKU1 (HCoV-HKU1), human coronavirus OC43 (HCoV-OC43), the Middle East respiratory syndrome-related coronavirus (MERS-CoV), and the severe acute respiratory syndrome coronavirus (SARS-CoV)) [1]. The HCoV (human coronavirus) is responsible for up to 10% - 30% of the upper respiratory tract infections globally [2]. Historically, HCoV’s were only responsible for mild infections until 2002, with the emergence of the severe acute respiratory syndrome (SARS) that started in the Guangdong province of China. In 2012, HCoV was responsible for an outbreak of another epidemic, MERS (Middle East respiratory syndrome).

The CoV-2 is an enveloped, single-stranded, positive-sense ribonucleic acid (RNA) virus. The virus has a spheric shape, 60 - 140 nm in diameter, surrounded by glycoprotein spikes (S) of 9 - 12 nm of length that gives them a crown-like appearance [3]. About 81 genomes of the SARS-CoV-2 has been isolated. The genome has approximately 27,000 to 32,000 bases in length and is encased in a nucleocapsid. It has four to five structural proteins: spike (S) protein, membrane (M) protein, nucleocapsid (N) protein, haemagglutinin-esterase fusion glycoprotein (HEF), and small envelope protein (E). The spike proteins allow the fusion with the host cell membrane. The nonstructural protein 15 (Nsp15) spike protein is identical in 89% to the other SARS- and MERS-CoV Nsp15s and has a high affinity to the human angiotensin-converting enzyme 2 (ACE2) receptors [4-5]. The nucleocapsid proteins hold the RNA genome and form the nucleocapsid. The hemagglutinin-esterase glycoprotein is found only in the beta coronaviruses, HCoV-OC43, and HKU1. The membrane and envelope proteins are responsible for the assembly and release of the virus. 

The most recent outbreak of the coronavirus (CoV) started in December 2019 in the Wuhan Hubei province in China and has so far affected 31 countries [6]. A large number of infected people from the Wuhan province were in contact with an animal market, which supports the possibility of a zoonotic origin.

HCoV spreads via respiratory transmission. Based on available data of SARS and MERS, the Centers for Disease Control and Prevention (CDC) has estimated an incubation period for the COVID-19 to be between two and 14 days [6]. The severity of symptoms depends on the patient's age and immune status [7]. There have been reported cases involving large populations showing people with varying incubation periods and the severity of symptoms based on age and immune status. The most common symptoms include fever, cough, and shortness of breath. Additional symptoms, such as fatigue, hemoptysis, and diarrhea, are also reported. The most common radiographic finding is diffuse bilateral ground-glass opacities found bilaterally and predominantly in the lower lobes. Common laboratory findings include leukopenia, lymphopenia, and thrombocytopenia with elevated C-reactive protein (CRP) levels [8]. 

## Case presentation

We hereby present the case of a 33-year-old man with a past medical history of alpha thalassemia who came to the emergency department (ED) with generalized weakness, fever, chills, productive cough with scant yellow sputum, myalgia, and arthralgia for 10 days. The patient endorsed a lack of appetite and an episode of non-bilious vomiting with epigastric pain and dysuria. At presentation, the patient denied chest pain, shortness of breath, palpitations, or diarrhea. The patient had not traveled outside of the United States (US) and had no known allergies. Physical examination was remarkable for scattered rhonchi bilaterally. The patient was not found to be in any distress. Heart sounds were normal. The patient was alert and oriented to time, place, and person. Cranial nerves, II-XII, were grossly intact, sensation was intact to touch, and strength was 5/5 throughout the upper and lower extremities bilaterally. In the ED, his vital signs included a temperature of 100.7°F, blood pressure (BP) of 105/54 mmHg, a mean arterial blood pressure of 71 mmHg, heart rate of 70 beats/minute, respiratory rate of 16/minute, and saturation of 97% on room air. Laboratory results were as follows: white blood count (WBC) 3.9 Lx10^3^/uL (normal 5.2 - 12.4 Lx10^3^/uL), a hemoglobin (Hgb) of 13.9 g/dL (normal: 12 - 18 g/dL), a hematocrit (Hct) of 42.9% (normal: 42% - 52%), and a platelet count of 176 x 10^3^/uL (normal: 130 - 400 x 10^3^/uL). C-reactive protein was 6.1 mg/dL (normal: 0.5 - 0.9 mg/dl), complement component (C3) 165 mg/dL (normal: 81 - 157 mg/dL), lactic acid 8.7 mg/dL (normal: 8.4 - 10.2 mg/dl), lactate dehydrogenase (LDH) 746 U/L (normal: 313 - 618 U/L), and procalcitonin < 0.05 ng/ml (normal: 0.00 - 0.05 ng/ml). 

**Figure 1:**
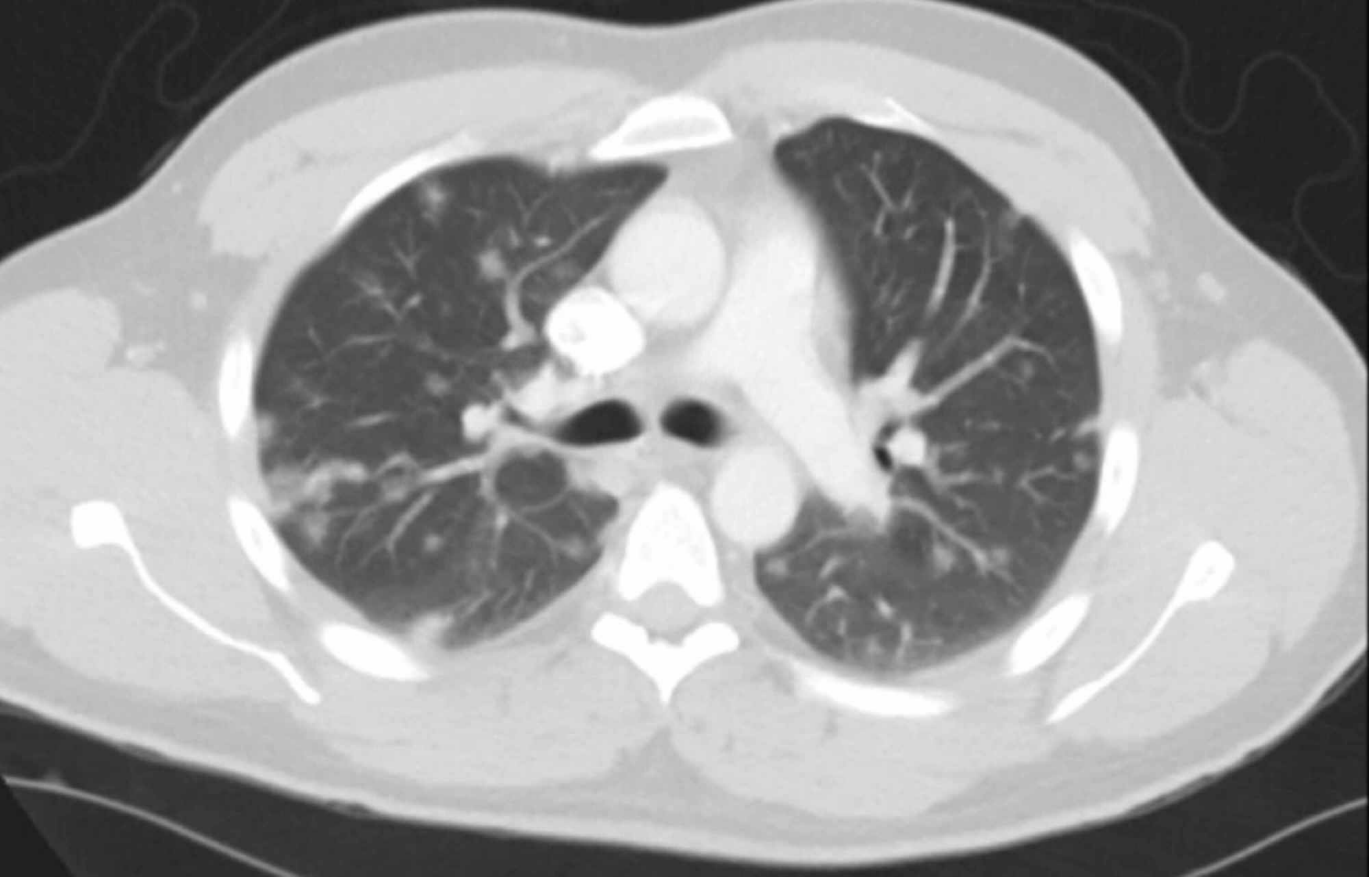
Axial computed tomography (CT) image of the chest showing bilateral ground-glass opacities

**Figure 2:**
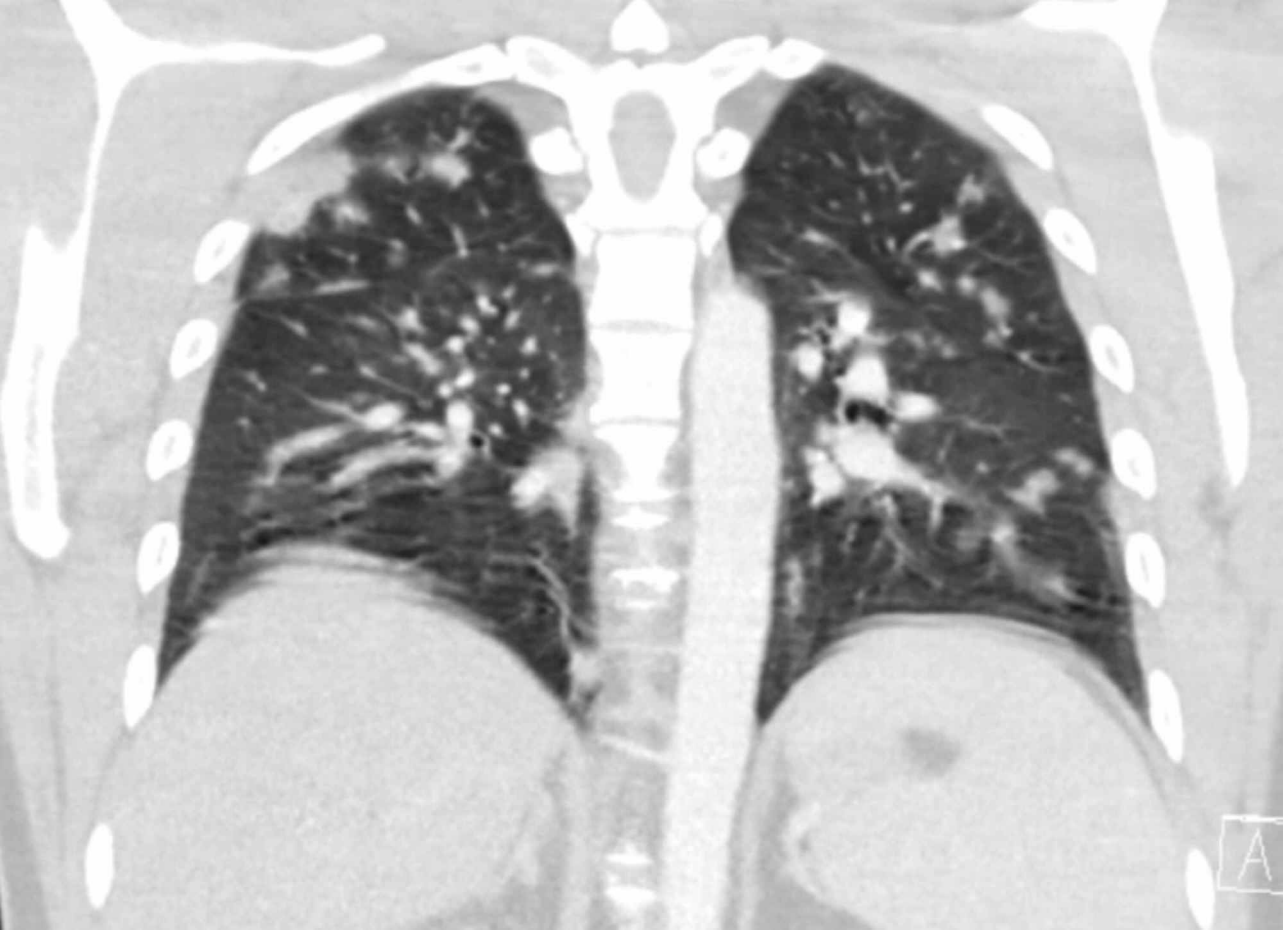
Coronal computed tomography (CT) image of chest showing bilateral ground-glass opacities

An echocardiogram (EKG) showed normal left ventricular systolic function. Left ventricular ejection fraction, estimated by two-dimensional (2D) EKG at 60% - 65%. Blood, urine, and sputum cultures were all negative. Chest x-ray showed bilateral ground-glass opacities in the lower lobes (Figures 1-2). An acid-fast bacillus smear was negative. The patient was placed on airborne, droplet, and contact isolation because of the high suspicion of coronavirus infection. The patient was empirically started on hydroxychloroquine and azithromycin. The pulmonologist was consulted. The screening test came out to be positive for COVID-19. The COVID-19 taskforce, New York City Department of Health (NYC DOH), and CDC COVID-19 protocol were in full effect. The plan was made to discharge the patient on self-quarantine for 14 days to limit transmission to others. 

## Discussion

The current understanding regarding transmission of the coronavirus to this day is still being researched. The suspected cause has been researched from the initial source of the outbreak in Wuhan, China. There was an association between the initial patients and a local seafood market which sold live animals. The patients were mostly workers or visitors to that area which has since been closed. However, the transmission of the virus outside of Wuhan has been more linked to person-to-person contact. This was suspected when reports of the virus were being made in places, such as Hubei and the US, where individuals did not have exposure to animal/seafood markets. Transmission by this method has been thought to be due to respiratory droplets from person to person. Close contact with infected individuals (within six feet) through respiratory droplets (coughing/sneezing) landing in the mouths/noses of people nearby or inhaled through the lungs are the suspected mode of transmission of the virus between people. In addition, there are still reported cases of the virus in locations where it is still not known how or where the individuals were exposed [9-11]. Though there have been reported cases in other countries initially attributed to those with travel to China or contact with travelers from China, there are ongoing local transmissions being found in areas where individuals had no contact with people traveling not only from China but other countries with known ongoing cases (Italy, Iran, Japan) [10]. In the US especially, there are known cases of COVID-19 in individuals who have not had travel or contact with anyone with known COVID-19, which raises the possibility of local transmission in these suspected areas. So far, several countries are still dealing with widespread ongoing transmission, some even with restrictions to entry to the US (China, Iran and Italy), while others are simply dealing with widespread transmissions or sustained community transmissions without restricted entry to the US [9-12]. Prevention is the key. All cases are reported to the CDC.

## Conclusions

COVID-19 can prove to be deadly in immunocompromised individuals and individuals with comorbidities; otherwise, the illness in healthy people is no more than the flu. Prevention is the key to avoiding crowded places and limiting exposure to others.
